# Recombinant probiotic *Lactococcus lactis* delivering P62 mitigates moderate colitis in mice

**DOI:** 10.3389/fmicb.2024.1309160

**Published:** 2024-04-04

**Authors:** Juliana Guimarães Laguna, Andria dos Santos Freitas, Fernanda Alvarenga Lima Barroso, Luís Cláudio Lima De Jesus, Octávio Augusto Greco Gomes De Vasconcelos, Ludmila Silva Quaresma, Monique Ferrary Américo, Gabriela Munis Campos, Rafael de Assis Glória, Joyce da Cruz Ferraz Dutra, Tales Fernando Da Silva, Kátia Duarte Vital, Simone O. Fernandes, Ramon O. Souza, Flaviano dos Santos Martins, Enio Ferreira, Túlio Marcos Santos, Alexander Birbrair, Marcos Felipe Andrade De Oliveira, Ana Maria Caetano Faria, Rodrigo Dias de Oliveira Carvalho, Franco Maria Venanzi, Yves Le Loir, Gwénaël Jan, Éric Guédon, Vasco Ariston de Carvalho Azevedo

**Affiliations:** ^1^Department of Genetics, Ecology, and Evolution, Federal University of Minas Gerais, Belo Horizonte, Brazil; ^2^Department of Clinical Analysis and Toxicology, Federal University of Minas Gerais Belo Horizonte, Minas Gerais, Brazil; ^3^Department of Microbiology, Federal University of Minas Gerais, Belo Horizonte, Minas Gerais, Brazil; ^4^Department of General Pathology, Federal University of Minas Gerais, Belo Horizonte, Minas Gerais, Brazil; ^5^Department of Dermatology, School of Medicine and Public Health, University of Wisconsin-Madison, Madison, WI, United States; ^6^Department of Biochemistry and Immunology, Federal University of Minas Gerais, Belo Horizonte, Minas Gerais, Brazil; ^7^School of Biosciences and Veterinary Medicine, University of Camerino, Matelica, Italy; ^8^STLO, INRAE, Institut Agro, Rennes, Brazil

**Keywords:** inflammatory bowel disease, probiotic bacteria, recombinant protein, immunomodulation, epithelial barrier, gut microbiota

## Abstract

**Introduction and objective:**

p62 is a human multifunctional adaptor protein involved in key cellular processes such as tissue homeostasis, inflammation, and cancer. It acts as a negative regulator of inflammasome complexes. It may thus be considered a good candidate for therapeutic use in inflammatory bowel diseases (IBD), such as colitis. Probiotics, including recombinant probiotic strains producing or delivering therapeutic biomolecules to the host mucosal surfaces, could help prevent and mitigate chronic intestinal inflammation. The objective of the present study was to combine the intrinsic immunomodulatory properties of the probiotic *Lactococcus lactis* NCDO2118 with its ability to deliver health-promoting molecules to enhance its protective and preventive effects in the context of ulcerative colitis (UC).

**Material and methods:**

This study was realized *in vivo* in which mice were supplemented with the recombinant strain. The intestinal barrier function was analyzed by monitoring permeability, secretory IgA total levels, mucin expression, and tight junction genes. Its integrity was evaluated by histological analyses. Regarding inflammation, colonic cytokine levels, myeloperoxidase (MPO), and expression of key genes were monitored. The intestinal microbiota composition was investigated using 16S rRNA Gene Sequencing.

**Results and discussion:**

No protective effect of *L. lactis* NCDO2118 pExu:*p62* was observed regarding mice clinical parameters compared to the *L. lactis* NCDO2118 pExu: *empty*. However, the recombinant strain, expressing p62, increased the goblet cell counts, upregulated *Muc2* gene expression in the colon, and downregulated pro-inflammatory cytokines Tnf and Ifng when compared to *L. lactis* NCDO2118 pExu: *empty* and inflamed groups. This recombinant strain also decreased colonic MPO activity. No difference in the intestinal microbiota was observed between all treatments. Altogether, our results show that recombinant *L. lactis* NCDO2118 delivering p62 protein protected the intestinal mucosa and mitigated inflammatory damages caused by dextran sodium sulfate (DSS). We thus suggest that p62 may constitute part of a therapeutic approach targeting inflammation.

## 1 Introduction

Inflammatory bowel diseases (IBD) constitute chronic or recurrent inflammatory ailments of the gastrointestinal tract (GIT). They constitute a growing public health challenge worldwide (Ng et al., 2017) and are divided into two types: Ulcerative Colitis (UC) and Crohn’s Disease (CD; Abraham and Cho, 2009; Glassner et al., 2020). Genetics and environmental factors such as antibiotic usage and the type of diet may alter immune responses and intestinal microbiota composition and participate in IBD etiology (Glassner et al., 2020).

UC treatments include topical or systemic 5-aminosalicylic acids (5-ASA), Azathioprine and 6-mercaptopurine immunomodulators, corticoids, and cyclosporine (Derikx et al., 2016). However, these current therapies have limited efficacy for many patients, leading to surgical intervention (Frei et al., 2012; Derikx et al., 2016). In this context, due to their anti-inflammatory and immunomodulatory properties, probiotic microorganisms have been considered a promising therapeutic tool for reducing IBD progression (Santos Rocha et al., 2014; Wang et al., 2018). Strong evidence of efficacy was proven for a probiotic product, VSL#3, in a meta-analysis of clinical trials (Sniffen et al., 2018). Wang et al. (2018) demonstrated that the administration of this probiotic cocktail of eight strains (e.g., *Lactobacillus acidophilus*, *Lactobacillus delbrueckii* subsp. *bulgaricus*, *Lacticaseibacillus casei*, *Lactiplantibacillus plantarum*, *Streptococcus thermophilus*, *Bifidobacterium breve*, *Bifidobacterium longum* subsp. *infantis* and *Bifidobacterium longum* subsp. *longum*) was able to reduce the tumor in azoxymethane/dextran sulfate sodium (AOM/DSS) induced mice ulcerative colitis-associated carcinogenesis model and to decrease the TNFα and IL6 levels in the colon tissue.

Probiotics are live microorganisms that benefit the host when ingested adequately (Hill et al., 2014). The beneficial effects of these microorganisms are attributed to their ability to regulate the intestinal microbiota, increase short-chain fatty acids (SCFA) production (e.g., acetate, propionate, and butyrate), inhibit the proliferation of pathogenic bacteria, modulate the systemic and local immune responses, and to reinforce epithelial barrier via stimulation of tight junctions proteins expression and of mucus-producing goblet cells (Jonkers et al., 2012; Bellavia et al., 2013; Derikx et al., 2016; Jakubczyk et al., 2020).

Many bacterial strains have been used as probiotics to mitigate colitis. This includes *Lacticaseibacillus rhamnosus* GG, *Escherichia coli* Nissle 1917 (Kruis et al., 2004; Zocco et al., 2006; Derikx et al., 2016), *Lactobacillus delbrueckii* (Santos Rocha et al., 2014), and *Lactococcus lactis* (Luerce et al., 2014; Wong et al., 2017). According to Plaza-Díaz et al. (2017), *Lacticaseibacillus casei* strain Shirota could modulate the immune system, increasing secretion of interleukin 4 (IL-4) production while reducing that of interferon-gamma (IFNγ), in cultured human dendritic cells. It further restored the normal stimulatory capacity of cells by reducing the expression of Toll-like receptors 2 (TLR2) and 4 (TLR4). The ability to induce immunomodulatory cytokines *in vitro* was indeed shown to correlate with that to mitigate colitis *in vivo* (Foligne et al., 2007).

In addition to wild-type probiotics, recombinant probiotic bacteria, aiming to express and deliver bioactive molecules with anti-inflammatory properties, is also being explored as a promising therapeutic approach in intestinal inflammatory conditions. These bioactive molecules include the surface layer protein B (SlpB) of *Propionibacterium freudenreichii* CIRM-BIA 129 (Belo et al., 2021), the 65 kDa heat shock protein (Hsp65) of *Mycobacterium leprae* (Gomes-Santos et al., 2017; Barroso et al., 2021), the antimicrobial pancreatitis-associated protein I (PAP; Carvalho et al., 2017) and the human cathelicidin (hCAP18; Noguès et al., 2022), among others.

One of the bioactive molecules that reportedly have immunomodulatory properties is the p62 protein, which is also named sequestosome 1 (SQSTM1), a multifactor molecule that is involved in many processes, including proliferation, apoptosis, inflammation, autophagy, and immune response (Pankiv et al., 2007; Komatsu et al., 2012; Vincent et al., 2020). This protein participates in physiological pathways related to different human diseases, such as neurodegenerative (Geetha et al., 2012), metabolic (insulin resistance and obesity; Long et al., 2017), osteoporosis (Krela-Kaźmierczak et al., 2016), IBD (Moscat and Diaz-Meco, 2012; Sabbieti et al., 2015; Vincent et al., 2020) and cancer (Venanzi et al., 2013, 2019). Ortiz-Masiá et al. (2014) observed increased levels of p62 in epithelial cells of IBD patients’ damaged mucosa and in mice treated with DSS, when compared to non-damaged mucosa. The authors suggested impaired epithelial autophagy is associated with human and murine colon injury. p62 protein is a well-known autophagy adaptor. It plays a role in selective autophagy activation. This essential scaffold can bind a variety of partner proteins and participate in biological signaling, innate immunity, apoptosis, and inflammatory response (Kim et al., 2019). Indeed, impaired autophagy promotes IBD, and defective autophagic flux with elevated p62 was observed in IBD tissues. Besides, p62 constitutes not only a cargo receptor for autophagy, but also a central signaling hub, linking several important pro- and anti-inflammatory pathways (Hennig et al., 2021). As an example, it restricts inflammasome signaling. Indeed, ablation of p62 expression restricts mitophagy and enhances NLRP3 inflammasome activation. Furthermore, p62 supports the autophagy-dependent degradation of ubiquitinated inflammasome proteins, such as NLRP3, ASC, and AIM2.

The anti-inflammatory property of recombinant p62 protein was proposed by Cecarini et al. (2020) in a mouse model of Alzheimer’s disease. The authors demonstrated that consumption of the recombinant lactic acid bacterium *L. lactis* MG1363 carrying the pExu vector harboring the p62 encoding gene (pExu:*p62*) improved memory, modulated the ubiquitin-proteasome system, and reduced oxidative process and neural inflammation in mice.

We have previously shown the anti-inflammatory properties of another *L. lactis* strain, NCDO2118, during the remission period of chemically induced colitis (Luerce et al., 2014). The objective of the present study was to combine the intrinsic immunomodulatory properties of *L. lactis* NCDO2118 with its ability to deliver p62 to enhance its protective and preventive effects in the context of UC.

## 2 Materials and methods

### 2.1 Bacteria culture conditions

*Lactococcus lactis* NCDO2118 strain was provided by (The National Collection of Dairy Organisms, now NCFB (q.v.) National Collection of Food Bacteria, originally at Shenfield, Berkshire, United Kingdom). This strain was transformed, either by the empty pExu plasmid or by the pExu:*p62* plasmid containing a eukaryotic expression cassette encoding the human p62 protein (pExu:*p62*), which was reported previously (Mancha-Agresti et al., 2017; Cecarini et al., 2020). Recombinant strains (*L. lactis* pExu: *empty* and *L. lactis* pExu:*p62*) were grown overnight without agitation at 30°C on M17 broth (Difco), supplemented with glucose 0.5% and erythromycin (125 μg/mL). Bacteria were centrifuged (3,000 x *g*, 10 min, RT), and the pellets were resuspended in 0.1 M Phosphate Buffered Saline (PBS). Doses were prepared daily (1×10^9^ CFU/mL) from fresh cultures.

### 2.2 Animals

Six weeks old C57BL/6 mice (male) were obtained from the Bioterism Center (CEBIO) at the Federal University of Minas Gerais (UFMG, Belo Horizonte, Brazil) together. Then, they were randomly split into six experimental groups (n = six animals per group). Before starting the experiment, all mice were weighed, and the body weight was similar between the groups (16–17 g). Mice were housed in polycarbonate-ventilated cages (3 per cage) under controlled room conditions: 12 h-light/dark cycles, temperature (25 ± 2°C). They had access to standard chow and drinking water *ad libitum*. The experiment design was approved by the Local Animal Experimentation Ethics Committee (CEUA-UFMG, Protocol n° 178/2022), and all procedures were conducted according to the Brazilian Society of Sciences in Laboratory Animals (SBCAL) (n.d.).

### 2.3 Experimental design

**Figure 1:**
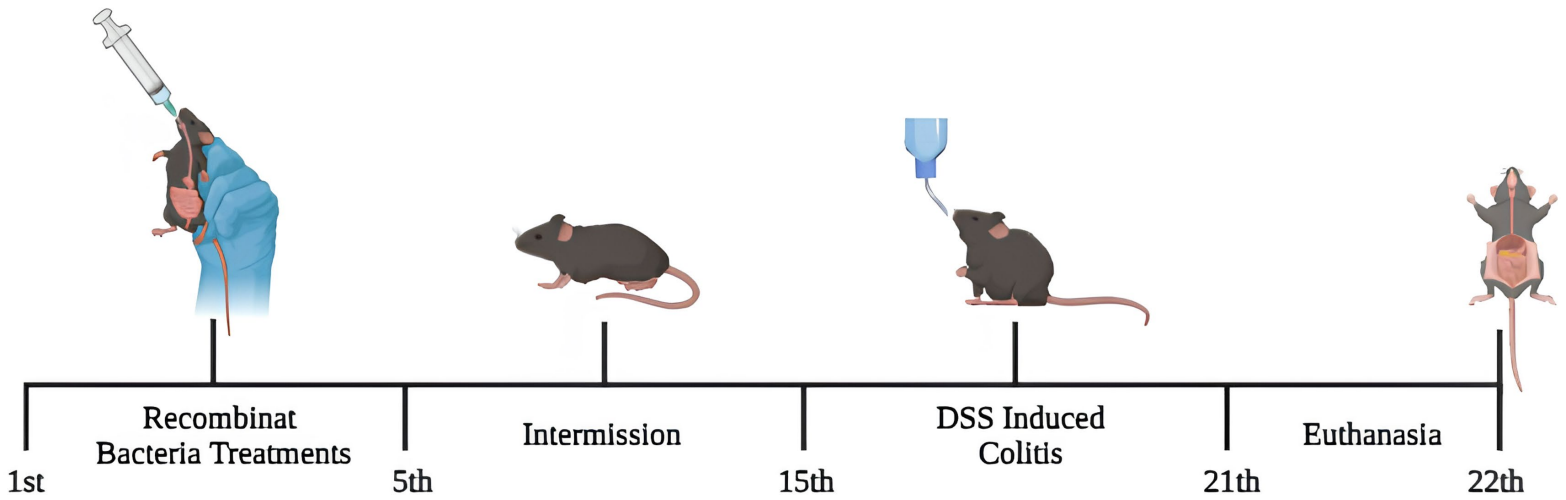
The experimental design. For 5 days, mice received treatments via gavage with recombinant *Lactococcus lactis* (pExu:*empty* or pExu:*p62*). After 10 days of intermission, mice were continuously inflamed by DSS 2% in drinking water and euthanized on the 22nd.

Mice were randomly split into three non-inflamed groups: Negative control (NC), *L. lactis* pExu: *empty* (pExu), *L. lactis* pExu:*p62* (P62), and three inflamed groups: Inflamed DSS 2% (DSS), *L. lactis* pExu: *empty* (DSS-pExu) and *L. lactis* pExu:*p62* (DSS-P62). During the experimental period, NC and DSS groups received 300 μL of phosphate-buffered saline (PBS, 0.1 M) solution daily by gavage for five consecutive days. In the same period, the other groups received daily treatments (300 μL) with *L. lactis* NCDO2118 (pExu: *empty* or pExu:*p62*; 10^9^ CFU/animal/day). After 10 days of intermission, DSS, DSS-pExu, and DSS-P62 groups were inflamed by the addition of DSS 2% in the drinking water (MP Biomedicals, Lot: S6132, Cat: 160110) provided *ad libitum* for 7 days. NC, pExu, and P62 groups received sterile water in the same period. Liquid and chow intake were measured during all experiment periods. On the 22^nd^ day, mice were anesthetized by a single intraperitoneal injection (16 mg/kg of xylazine and 80 mg/kg of ketamine, Syntec, Tamboré, Brazil; *i.p*) for blood collection via an axillary vein. Afterward, the animals were euthanized by cervical dislocation, and liver, spleen, and colon tissues were harvested and stored immediately at −80°C (Figure 1).

### 2.4 Intestinal permeability

Intestinal permeability was analyzed by determination of radioactivity in the blood. For that, 4 h before the procedures of euthanasia, all mice received, via gavage, 100 μL containing 18.5 MBq of Diethylenetriaminepentaacetic acid (DPTA) labeled with ^99m^technetium (^99m^Tc-DTPA) to evaluate the impact of recombinant p62 protein on the epithelial barrier (Carvalho et al., 2021). After this period, mice were anesthetized, and 300 μL of blood was collected. According to the manufacturer’s instructions, the radioactivity was measured by a gamma radiation counter (Wizard, PerkinElmer). Data was expressed in dose percentage (%) of ^99m^Tc-DTPA/g, according to the equation below:


$$\%\;\text{of}\;\text{marker}=\frac{\text{blood}\;\text{cpm}}{\text{administrated}\;\text{dose}\;\text{of}\;99\text{mTc}-\text{DTPA}\;\text{cpm}}\;x\;100$$

cpm = count per minute.

### 2.5 Myeloperoxidase enzyme activity

Neutrophils’ recruitment to intestinal mucosa was evaluated by measuring the MPO enzymatic activity in colonic extracts (Barroso et al., 2021). Briefly, colon tissue was homogenized with PBS 0.1 M and centrifuged at 3,000 x *g* for 10 min at 4°C. Afterward, pellets hypotonic lysis was performed with a buffer containing 0.5% hexadecyltrimethylammonium bromide (Sigma-Aldrich) with three freeze–thaw cycles in liquid nitrogen. After centrifugation (3,000 x *g*, 15 min, 4°C), the supernatant was collected for colorimetric assay using 1.6 mM 3,3,5,5′-Tetramethylbenzidine (Sigma-Aldrich) as a chromogenic enzyme substrate. The absorbance was measured at 450 nm (MPO) on a microplate spectrophotometer (Bio-Rad 450 model, Bio-Rad Laboratories). The result was expressed as MPO arbitrary units/mg of tissue.

### 2.6 Secretory IgA dosage in the intestinal fluid

Secretory IgA total levels were analyzed by enzyme-linked immunosorbent assay (ELISA), according to Martins et al. (2009). Small intestine content was removed, weighed and intestinal secretion was washed out using 2 mL of 0.1 M PBS (pH 7.2) supplemented with protease inhibitor (1 μM Aprotinin; 25 μM Leupeptin; 1 μM Pepstatin; 1 mM Phenylmethylsulfonyl fluoride-PMSF). Samples were centrifuged at 2000 rpm for 30 min at 4°C; the supernatant was stored at −80°C until immunoglobulin dosage. The ELISA assay was performed in Nunc-Immuno MaxiSorp microtiter plates using goat anti-mouse IgA antibody (M-8769, Sigma, St. Louis, United States) in coat buffer (1 M Na2CO3; 0.1 M NaHCO3; pH 9.6) for 18 h at 4°C, washed (0.1 M PBS + 0.05% Tween 20), and blocked (1% albumin in PBS-Tween 20). Afterward, pre-diluted intestinal fluids (1:1000 in 0.1 M PBS-Tween 20) were incubated for 1 h. The plates were then washed, and a biotin-conjugated anti-mouse IgA antibody (dilution 1:1000, A4789, Sigma-Aldrich, St. Louis, MO, United States) was added and incubated for 1 h. Finally, 100 μL/well of OPD (1 mg/mL) and 0.04% H_2_O_2_ substrates were added and set for 10 min. The reaction was stopped with 20 μL/well of 1 M H2SO4 (Sigma-Aldrich, St. Louis, MO, United States). The assay reaction absorbance was measured at 492 nm using a microplate reader (Bio-Rad Laboratories, Hercules, CA, United States). Results were expressed in intestinal content’s sIgA concentration (μg/mL).

### 2.7 Cytokine concentration

Samples (100 mg) of colon tissue were weighed and homogenized in 500 ul of cytokine extraction solution (2 μL of Aprotinin (Sigma Aldrich), 500 mg of Bovine serum albumin (BSA), 4.40 mg of Benzethonium chloride (Sigma Aldrich), 2.34 g of Sodium Chloride (NaCl), 100 μL of DMSO, 37.2 mg of EDTA, 1.7 mg of PMSF in 100 mL of 0.1 M PBS plus 50 μL of Tween 20) using the Precellys tissue homogenizer and then samples were frozen at −80°C. IL10, IL6, and TGFβ cytokines concentrations were analyzed by enzyme-linked immunosorbent assay (Elisa), according to fabricant recommendation (DuoSet^®^ Elisa Development System).

### 2.8 Histological analysis

After euthanasia, the liver, spleen, and entire colon were removed, and colon length was measured. For histological analysis, the liver, spleen, and colon were washed with PBS 0.1 M, and the proximal portion of the colon was rolled up. After, the tissues were immersed in a 10% buffered formaldehyde solution (Labsynth, São Paulo, Brazil) for fixation. Tissues were embedded in paraffin, and 4 μm thick slices of samples were placed on a glass slide and stained with hematoxylin and eosin (HE) or periodic acid–Schiff (PAS). The intestinal histological inflammation score was determined as described by Erben et al. (2014). Ten colon slide images from each animal were captured by a BX41 optical microscope (Olympus, Tokyo, Japan) for morphometric examination, and the crypt depth was measured. These analyses were performed using *ImageJ* 1.51j.8 software (NIH, Bethesda, MD, United States). The spleen and liver histological scores were analyzed according to Cesta (2006) and Liu et al. (2022), respectively (Supplementary Figure S1).

### 2.9 Mice colon relative gene expression analysis

Mice colon slices were collected during the euthanasia procedures and stored at −80°C. According to the recommended protocol, total RNA isolation of the colon was carried out using Pure Link™ RNA Mini Kit (Invitrogen). The samples were analyzed by NanoDrop 2000 spectrophotometer to verify the total RNA concentration and quality (Thermo Scientific; 260/280 ratio: 2.0–2.2). Also, RNA integrity was verified in 1.5% agarose gel. Residual genomic DNA was removed using the Turbo DNA-free^™^ Kit (Invitrogen). Total RNA was used to make 1,000 ng of complementary DNA (cDNA) by High-Capacity cDNA Reverse Transcription kit (Applied Biosystems^™^, ThermoFisher), according to recommended instructions. Quantitative PCR was carried out with the PowerUp^™^ SYBR^®^ Green Master Mix (ThermoFisher) on the ABI PRISM 7900HT Sequence Detection System (Applied Biosystems^™^) under the following steps: 95°C for 10 min and 40 cycles of 95°C for 15 s, and 60°C for 1 min. Glyceraldehyde dehydrogenase (*Gapdh*) and β-actin *(Actb)* were used as housekeeping genes. Mucin 2 *(Muc2)*, Zonulin *(Hp)*, and Occluding (*Ocln*) were used as intestinal barrier markers. Also, Interleukins 1β (*Il1b*), 17A *(Il17a)*, Tumor necrosis factor *(Tnf)*, Interferon-gamma (*Ifn*g), and Transforming growth factor beta 1 (*Tgfb1*) were used as inflammatory cytokines (Table 1). Gene expression results were analyzed following the 2^−ΔΔCT^ method.

**Table 1:** Sequence of primers analyzed by RT-qPCR assay.

Genes	Primer forward	Primer reverse	Reference
*Actb*	GCTGAGAGGGAAATCGTGCGTG	CCAGGGAGGAAGAGGATGCGG	Volynets et al. (2016)
*Gapdh*	TCACCACCATGGAGAAGGC	GCTAAGCAGTTGGTGGTGCA	Giulietti et al. (2001)
*Muc2*	GATGGCACCTACCTCGTTT	GTCCTGGCACTTGTTGGAAT	Volynets et al. (2016)
*Hp*	CCACCTCTGTCCAGCTCTTC	CACCGGAGTGATGGTTTTCT	Volynets et al. (2016)
*Ocln*	ACTCCTCCAATGGACAAGTG	CCCCACCTGTCGTGTAGTCT	Volynets et al. (2016)
*Il1b*	CTCCATGAGCTTTGTACAAGG	TGCTGATGTACCAGTTGGGG	Song et al. (2013)
*Il17a*	GCTCCAGAAGGCCCTCAGA	AGCTTTCCCTCCGCATTGA	Giulietti et al. (2001)
*Tnf*	ACGTGGAACTGGCAGAAGAG	CTCCTCCACTTGGTGGTTTG	Song et al. (2013)
*Ifn*γ	TCAAGTGGCAATAGATGTGGAAAGAA	TGGCTCTGCAGGATTTTCATG	Machain-Williams et al. (2013)
*Tgfb1*	TGACGTCACTGGAGTTGTACGG	GGTTCATGTCATGGATGGTGC	Giulietti et al. (2001)

### 2.10 DNA sequencing

Mice stools were collected during the 21°day of the experimental period and stored at - 80°C for future analyses. For that, animals were separated, and the collection was done according to the natural physiology of each animal. The DNA from mice stool was extracted according to Yu and Morrison (2004) with modifications. Samples were sent to Neoprospectra Microbiome Technologies-Brazil, and they processed the next steps. The primer sequence used in this study was 341F (CCTACGGGRSGCAGCAG) and 806R (GGACTACHVGGGTWTCTAAT). The libraries were prepared using the TruSeq Library Prep kit (Illumina, United States) for sequencing bacterial amplicons using oligonucleotides 341F and 806R specific for the V3/V4 region of the 16S rRNA (Caporaso et al., 2012). Libraries were sequenced on the MiSeq system (Illumina, United States). Paired reads of 500 cycles were performed using the V3x600 sequencing kit (Illumina, United States) with 100,000 times per sample coverage.

### 2.11 Bioinformatic analysis

The Bioinformatics analysis for taxonomic classification was performed as follows. Quality filters were applied to fastq files, including the removal of truncated and low-quality readings (Phred score < 20) using the Trimmomatic tool (Bolger et al., 2014). Then, sense and antisense paired reads were merged into contigs. The sequences were subjected to singleton and chimera removal, and subsequently, the sequences were grouped into Taxonomic Operational Units (OTUs) using Uchime v. 4.2.40 and Vsearch v. 2.22.1 (Edgar et al., 2011; Rognes et al., 2016) and assigned taxonomically, considering a 97% similarity against sequences from the SILVA database (Quast et al., 2013). The Shannon index was employed to estimate alpha diversity using the RStudio packages Vegan, Fossil, and Microbiome. Shannon (H′) index is expressed as H′ = −Σni/n ln (ni/n), where ni represents the number of individuals in taxon i, and n is the total number of individuals. This index, serving as a measure of heterogeneity, considers species richness and evenness. The evenness of species diversity was computed using the Pielou formula: H′/H′max, where H′ is the Shannon index, and H′max is the maximum potential diversity of the number of species (S) within the community, determined by H′max = ln S (Shannon, 1948). Regarding beta diversity, PCoA analyses were conducted using the Bray–Curtis distance matrix to investigate group differences. The relative abundance of OTU across the groups was determined using STAMP software (Parks et al., 2014).

### 2.12 Statistical analysis

The results are presented as the mean ± standard error (SE). The Kolmogorov–Smirnov test was performed to determine the normality of distribution. Parametric data were evaluated by one-way ANOVA followed by the Tukey post-test. The normal distribution of OTU data was validated using the Shapiro–Wilk Test. A one-way ANOVA followed by the Tukey *post-hoc* test was applied to assess the variance in mean values across various group comparisons. In the case of multivariate data, distinctions among groups were identified using ANOSIM. Statistical significance was established with a value of p below 0.05. Data were analyzed using GraphPad Prism 8.0 software (GraphPad Software, San Diego, California, United States), RStudio v 4.2.1, and STAMP v. 2.1.3.

## 3 Results

### 3.1 p62 protein attenuates colon length shortening

**Figure 2:**
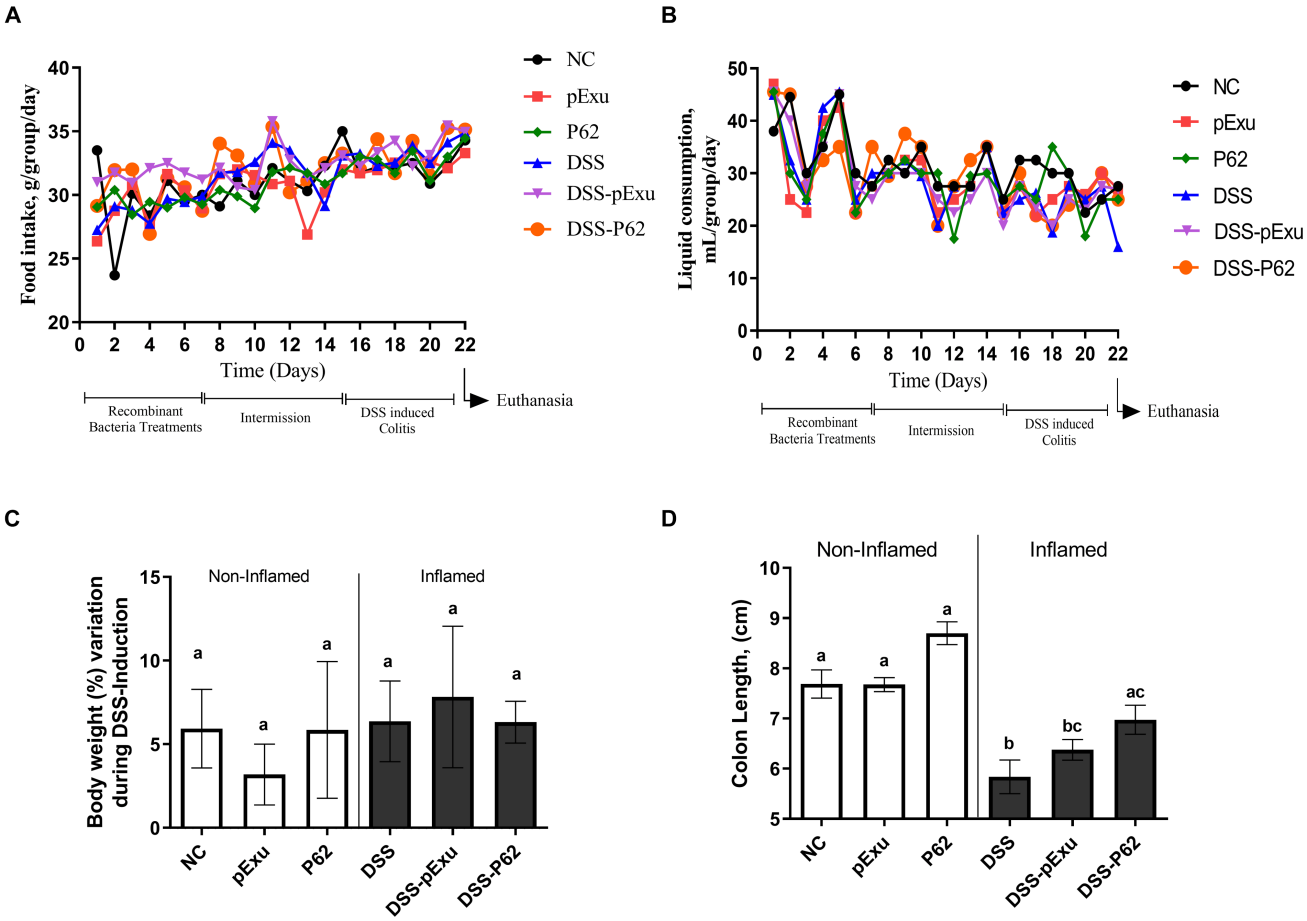
Effect of p62 protein on mice clinical parameters and colon length. Analysis of food **(A)** and liquid intake **(B)**, body weight loss **(C)**, and colon length **(D)** in mice inflamed with DSS 2%. Means ± SE followed by different lowercase letters indicate a statistically significant difference (*p* < 0.05) assessed by one-way ANOVA to multiple comparisons among treatments, followed by Tukey’s test.

Food intake and liquid consumption were analyzed daily. There was no statistically significant difference regarding food and liquid consumption among the groups during the experimental period (*p > 0.05*; Figures 2A,B). In addition, no alteration was observed in the body weight among the groups (*p > 0.05*; Figure 2C). Moderate DSS-induced colitis triggered colon length shortening. Indeed, mice in the DSS group presented a shorter colon length (5.83 ± 0.33 cm) when compared to the control (NC; 7.68 ± 0.28; *p < 0.05*; Figure 2D). *L. lactis* NCDO2118 pExu:*empty* consumption did not mitigate this shortening, as the colon length was similar in the DSS-pExu (6.37 ± 0.20) and the DSS groups. However, consumption of *L. lactis* NCDO2118 pExu:*p62* attenuated the colon length shortening (6.97 ± 0.29) when compared to the DSS group (5.83 ± 0.33; *p < 0.05*).

### 3.2 p62 Protein expression does not improve *L. lactis* ability to prevent colon mucosa damage

**Figure 3:**
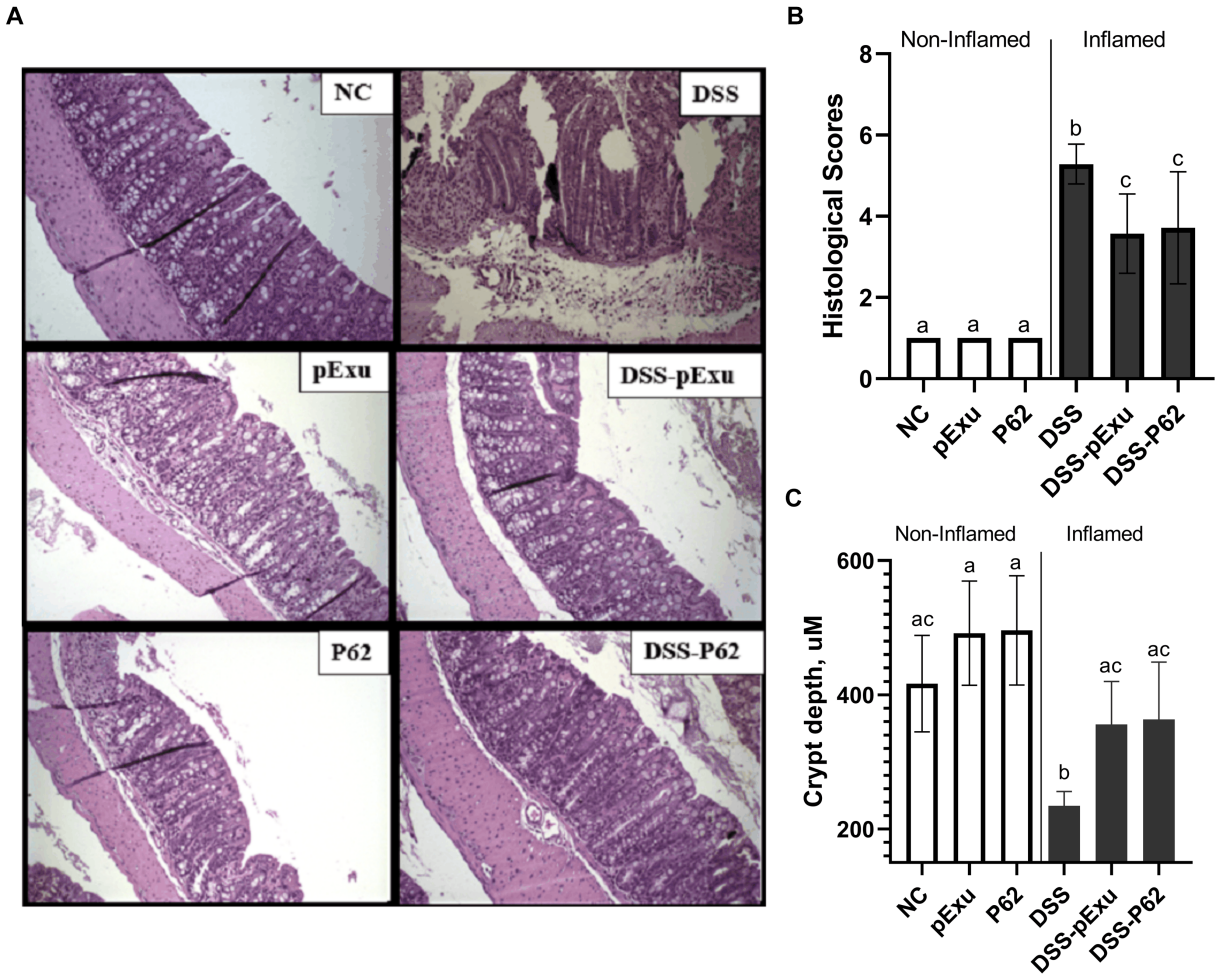
p62 protein Does not improve intestinal mucosa damage induced by DSS 2%. **(A)** Colon mucosa histopathology **(B)** histopathological score; and **(C)** crypt depth. Means ± SE followed by different lowercase letters indicate a statistically significant difference (*p* < 0.05) assessed by one-way ANOVA to multiple comparisons among treatments, followed by Tukey’s test.

Histological analysis showed that mice inflamed with DSS presented higher histopathological scores (Figure 3B) and lower crypt depth compared to the NC group (*p = 0.003*; Figure 3C) than healthy control mice. These alterations were mainly characterized by inflammatory cell infiltration into the lamina propria and damage to muscle extension. Both inflamed groups consuming *L. lactis* NCDO2118 pExu:*empty* or *L. lactis* NCDO2118 pExu:*p62* (DSS-pExu and DSS-P62) have ameliorated the histopathological scores and crypt depth when compared to DSS group. However, no difference was observed between the two groups receiving *L. lactis* strains (Figures 3A–C). Regarding the liver and spleen, no significant histopathological alterations were observed in these organs among the groups (*p > 0.05*; Supplementary Figure S1).

### 3.3 p62 protein increased goblet cells number and *Muc2* gene expression

The protective effect of *L. lactis* NCDO2118 pExu:*p62* toward the epithelial barrier was evaluated by monitoring intestinal permeability, goblet cell count, and expression of genes encoding mucin 2 and tight junction proteins.

**Figure 4:**
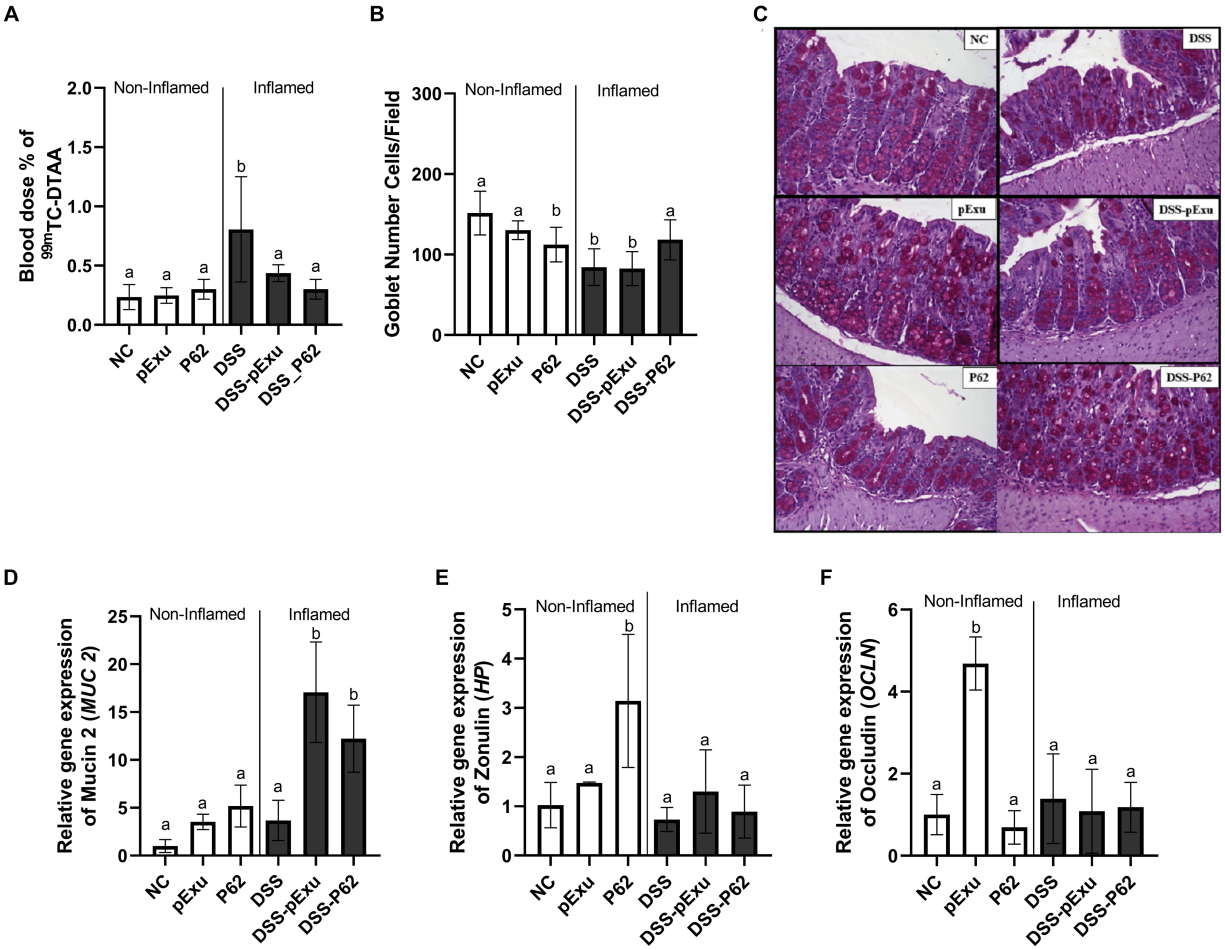
Protective effect of recombinant p62 protein on epithelial barrier. Intestinal permeability **(A)**; The number of goblet cells/field **(B)**; Representative photomicrographs from colon section stained with Periodic Acid-Schiff (PAS) **(C)**; and level of mRNA transcript levels of mucin 2 (*Muc2*) **(D)**, Zonulin (*Hp*) **(E)**, Occludin (*Ocln*) **(F)**. Means ± SE followed by different lowercase letters indicate a statistically significant difference (*p* < 0.05) assessed by one-way ANOVA followed by Tukey’s test.

DSS-induced mice colitis group (DSS) exhibited a significant increase in intestinal permeability (0.8064 ± 0.44% ID/g; 84.40 average of goblet cells) and a decrease in goblet cells number when compared to the control group (0.235 ± 0.04 %ID/g; 151.5 average of goblet cells; *p = 0.003*; Figures 4A,B). Consumption of *L. lactis* NCDO2118 pExu:*empty* (0.437 ± 0.07% ID/g; *p = 0.014*) and of *L. lactis* NCDO2118 pExu:*p62* both reduced the intestinal permeability (0.300 ± 0.08% ID/g; *p = 0.0004*) when compared to DSS group (Figure 4A). An increase was observed in the goblet cell count (118.3 average of goblet cells) when comparing the DSS-P62 group with other inflamed groups (Figures 4B,C).

Gene expression of markers associated with the epithelial barrier was also evaluated. For the non-inflamed and DSS group, no statistical differences were observed in the *Muc2* (1.00 ± 0.27 vs. 3.68 ± 0.93) gene expression (*p > 0.05*; Figure 4D). However, upregulation of *Hp* and *Ocln* mRNA transcripts was observed for non-inflamed P62 and pExu groups, respectively, compared to other groups (Figures 4E,F). High transcript levels of *Muc2* gene expression for the inflamed groups DSS-pExu (17.08 ± 1.71, *p < 0.0001*) and DSS-P62 (12.21 ± 1.56, *p = 0.0009*) were observed compared to the NC (1.00 ± 0.27) and DSS (3.68 ± 0.93) groups (Figure 4D).

### 3.4 p62 protein reduced inflammatory infiltration and modulated *Tnf* and *Ifn*g gene expression

**Figure 5:**
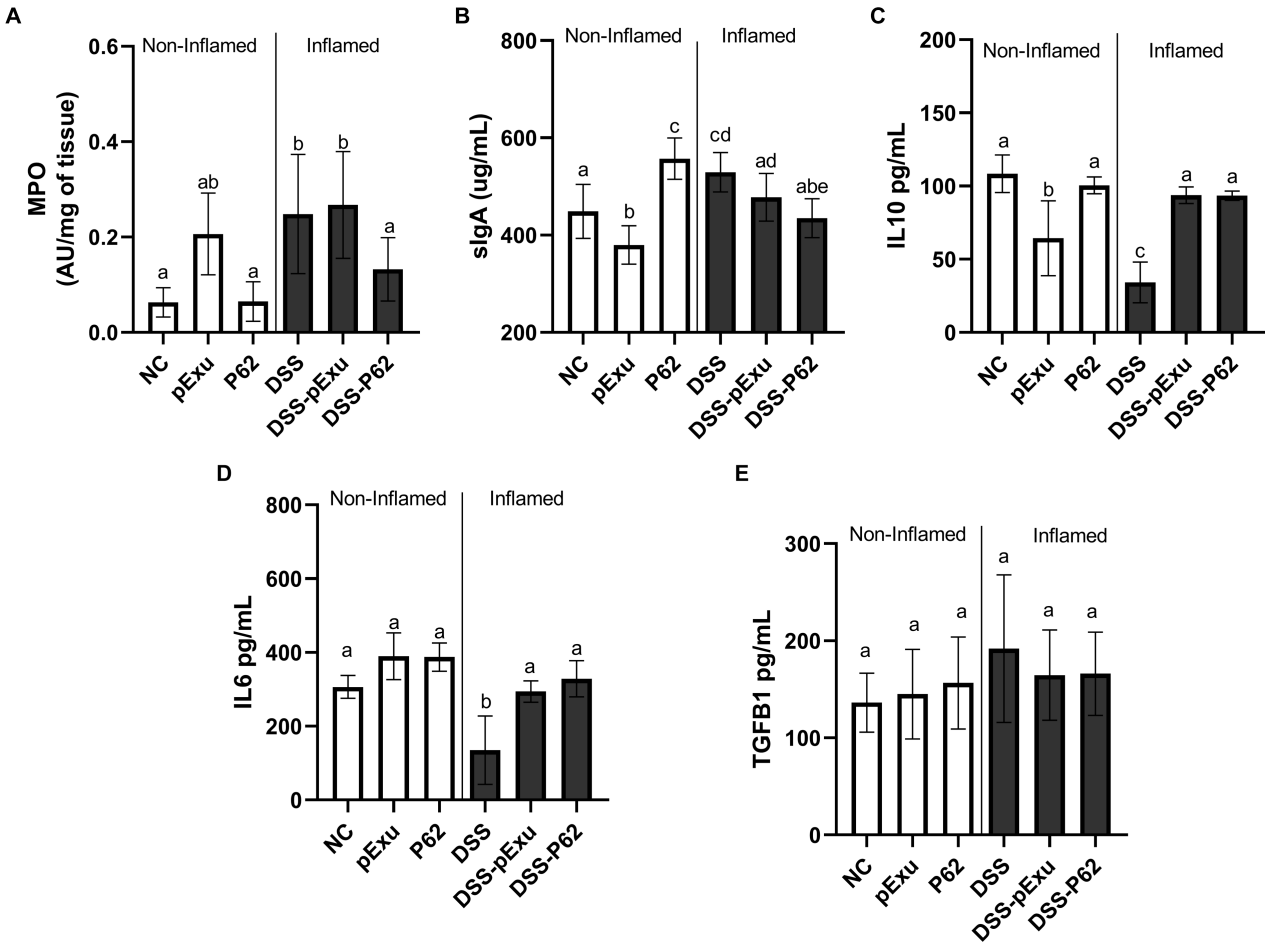
Effect of recombinant p62 protein on inflammatory markers. **(A)** Myeloperoxidase activity; **(B)** sIgA levels and total protein pg./mL of IL10 **(C)**, IL6 **(D)**, and TGFβ **(E)**. Means ± SE followed by different lowercase letters indicate a statistically significant difference (*p* < 0.05) assessed by one-way ANOVA to multiple comparisons among treatments, followed by Tukey’s test.

Inflammatory infiltration by neutrophils in the colon was assessed by monitoring the MPO enzyme activity. High levels of MPO enzyme activity were observed in the DSS (0.0248 ± 0.05; *p = 0.0077*) and DSS-pExu (0.2672 ± 0.04; *p = 0.0027*) groups when compared to the NC group (0.0630 ± 0.01; Figure 5A). In contrast to the other inflamed groups, consumption of *L. lactis* NCDO2118 pExu:*p62* prevented this increase. Indeed, the activity in the DSS-P62 group (0.1322 ± 0.04) was lower than in the DSS-pExu group and close to that in the control NC group (*p > 0.05*).

Regarding sIgA levels in the intestinal fluid, a high concentration of this immunoglobulin was measured in the inflamed group (DSS; 529.4 ± 15.28 μg/mL), compared to the NC group (448.82 ± 20.98 μg/mL; *p = 0.019*; Figure 5B). Also, compared to the NC group, sIgA secretion was lower in the pExu group (379.6 ± 13.06 μg/mL; *p = 0.041*) and higher in the P62 (557.2 ± 16.06 μg/mL; *p = 0.0007*) groups. In the context of colitis, the increase in sIgA level was prevented by the consumption of *L. lactis* NCDO2118 pExu:*p62* (DSS-P62; 434.7 ± 15.18 μg/mL), yet not by consumption of *L. lactis* NCDO2118 pExu:*empty* (Figure 5B).

The concentrations of pro-inflammatory (IL6 and TGFβ) and anti-inflammatory (IL10) cytokines were determined to evaluate the immunoregulatory effects of recombinant *L.* strains in the context of DSS-induced colitis in mice.

IL10 (34.18 pg./mL) and IL6 (135 pg./mL) cytokine concentrations were lower in the DSS group compared to the NC group (108 and 306 pg./mL, *p < 0.05*; Figures 5C,D). Recombinant *L. lactis* strains (DSS-pExu and DSS-P62 groups) increased the IL10 (93.73 vs. 93.44 pg./mL) and IL6 (294.2 vs. 328.6 pg./mL) cytokines concentration, compared to the DSS group (34.10 and 135 pg./mL, respectively; *p < 0.05*). There was no significant difference among groups regarding TGFβ cytokine concentration (Figure 5E). However, when using transcriptomics, it was observed that *Tgfb1* mRNA levels were lower in the non-inflamed P62 group, as well as in DSS-pExu and DSS-P62 groups, when compared to the colitis DSS group (Figure 5E; *p < 0.05*).

The expression of pro-inflammatory cytokines genes (*Tnf*, *Ifn*g, *Il17a, Tgfb1*, and *Il1b*) was also monitored further to evaluate the immunoregulatory effects of recombinant *L. lactis* strains.

**Figure 6:**
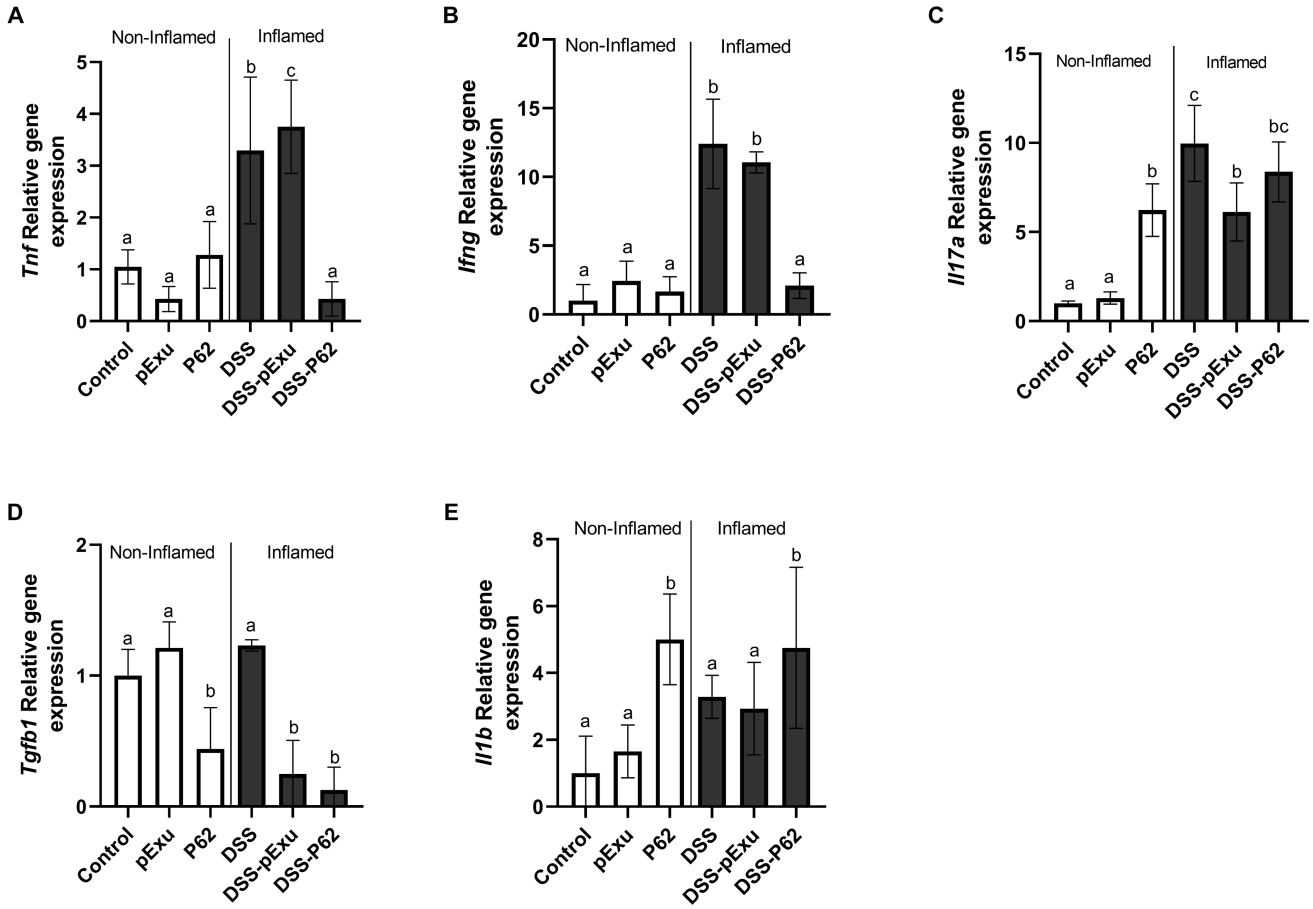
Effect of recombinant p62 protein on inflammatory markers. **(A)** Tnf, **(B)** *Ifn*g, **(C)** Il17a, **(D)** Tgfb1, and **(E)** Il1b. Means ± SE followed by different lowercase letters indicate a statistically significant difference (*p* < 0.05) assessed by one-way ANOVA to multiple comparisons among treatments, followed by Tukey’s test.

The DSS treatment triggered upregulation of *Tnf* (3.29 ± 0.63), *Ifn*g (12.41 ± 1.32), and *Il17a* (9.97 ± 0.93) gene expression when compared to the control NC group (1.00 ± 0.11; Figures 6A–C). There was no significant difference between the DSS-pExu and DSS-P62 groups regarding the *Il17a* and *Tgfb1* mRNA transcript levels (Figures 6D,E). However, genes were downregulated in the DSS-P62 (*p < 0.05*; Figures 6A,B). When compared to the DSS group, gene expression of *Tnf* (0.42 ± 0.14), *Ifn*g (11.06 ± 0.34 vs. 2.08 ± 0.41) and *Tgfb1* (1.23 ± 0.04 vs. 0.12 ± 0.17) was downregulated, and that of *Il1b* (4.75 ± 0.89) was upregulated in the DSS-P62 group (*p < 0.05*; Figures 6A–E).

### 3.5 p62 protein did not modulate the intestinal microbiota

**Figure 7:**
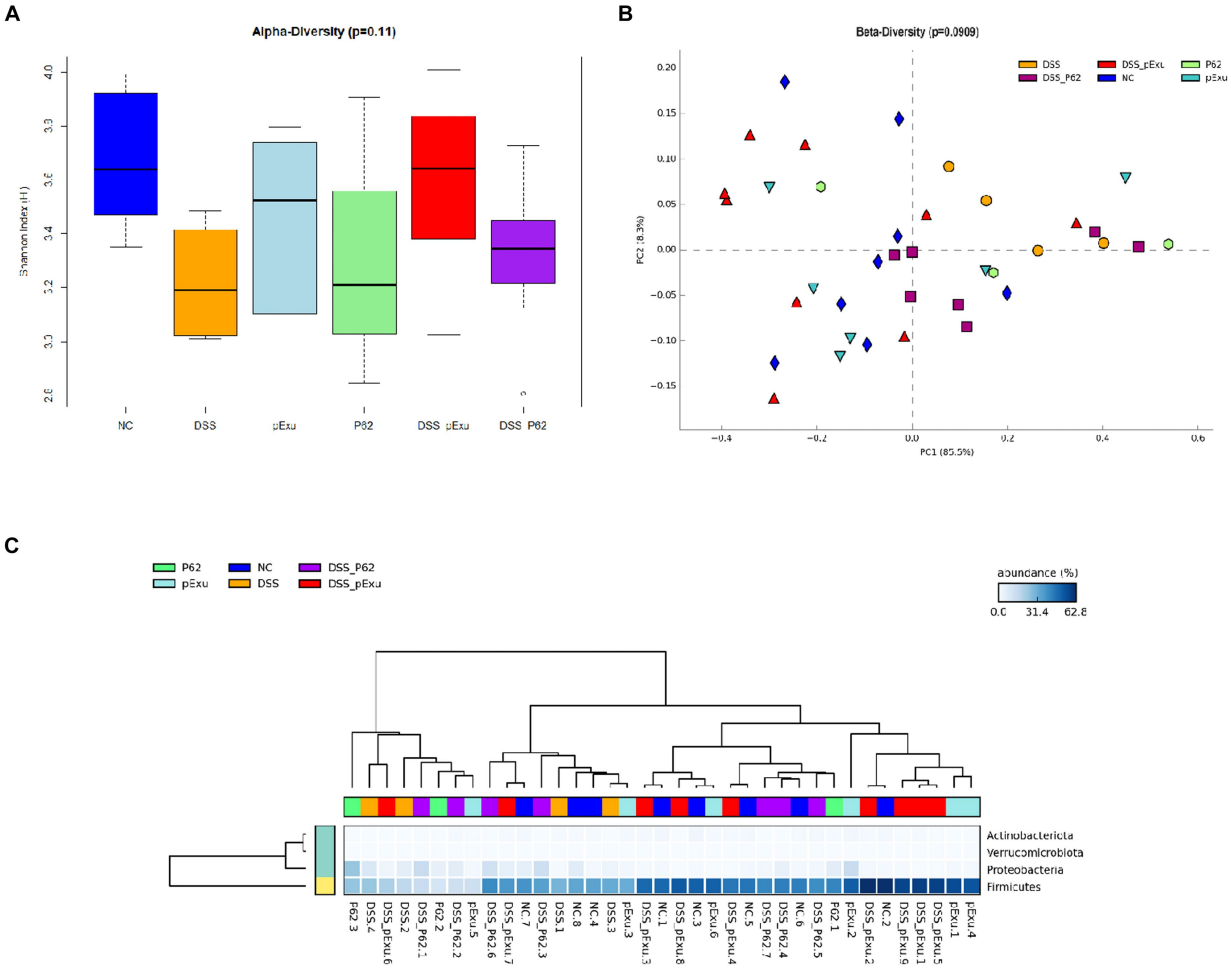
Effect of recombinant p62 protein on intestinal microbiota. **(A)** Shannon index, **(B)** Principal component analysis (PCA), and **(C)** Heatmap of bacterial taxon relative abundance. Data in the graph represent the proportion of reads assigned to a given phylum-level taxon. The dendrogram represents a hierarchical clustering based on the Unweighted Pair Group Method using the Arithmetic averages method.

Intestinal microbiota biodiversity was evaluated in this study. No difference was observed between the groups regarding alpha-diversity (ANOVA, *p* = 0.11) and beta-diversity (ANOSIM, *p* = 0.090). The proportion of Actinobacteriota and Firmicutes phyla population was decreased in the DSS group when compared to the negative control, while the treatment of mice receiving DSS with *L. lactis* harboring pExu empty vector restored the population of Firmicutes (*p* = 0,00146). At the genus level, the population of *Anaerotruncus*, *Colidextribacter*, *Enterorhabdus*, *Eubacterium* (Brachy group), *Lachnoclostridium*, UCG-001 (*Lachnospiracea*) and *Tuzzerella* were found decreased in the DSS control group, when compared to healthy mice. The administration of *L. lactis* harboring pExu empty vector significantly increased all these Genera, except for UCG-001 (*Lachnospiracea*). The treatment with *L. lactis* harboring p62 did not affect the mice’s gut microbiota composition (Figure 7).

## 4 Discussion

IBD constitutes a growing concern and encompasses diseases that affect people worldwide, with a higher occurrence in industrialized countries. IBD occurrence has been increasing with increasing consumption of fast food, yet decreasing consumption of fibers, with alteration of the immune system, gut microbiota, and intestinal permeability (Durchschein et al., 2016; Glassner et al., 2020). According to Kaplan (2015), the data might be underestimated due to people’s limited access to information, diagnostics, and adequate treatments.

Probiotics, prebiotics, symbiotics, paraprobiotics, and postbiotics constitute promising therapeutic alternatives to ameliorate IBD (Martyniak et al., 2021). Selected strains of probiotic bacteria have already been demonstrated to modulate the immune response, intestinal barrier, and permeability *in vivo* (Bellavia et al., 2013; Batista et al., 2020; Jakubczyk et al., 2020; Wei et al., 2022). Among them, *Lactococcus lactis* is of great interest for probiotic applications. Indeed, this lactic acid bacterium has a long history of safe use in producing fermented foods. According to its Generally Recognized as Safe (GRAS) status under United States Food and Drug Administration (FDA) regulations, its applications can be regarded. *L. lactis* strain NCDO2118, notably, revealed immunomodulatory properties *in vitro* in Caco-2 human intestinal epithelial cells (Luerce et al., 2014), as well as protective properties *in vivo* in a chronic colitis mouse model (Alves et al., 2023). *L. lactis* can be used as a tool to deliver therapeutic molecules aiming at the modulation of the immune system. Indeed, numerous studies have examined *in vivo* the potential of *L. lactis* as a delivery vector of heterologous proteins for vaccination purposes (Levit et al., 2022) or for treating IBD and mucositis (Carvalho et al., 2017).

The objective of the present study was to combine the intrinsic immunomodulatory properties of *L. lactis* NCDO2118 with its ability to deliver health-promoting molecules to enhance its protective and preventive effects in the context of UC. Several studies already evidenced the interest in using this strain to deliver *in vivo* therapeutic proteins in gut inflammation. It was shown that NCDO2118 expressing mycobacterial Hsp65 reduced intestinal inflammation and fibrosis in the TNBS-induced chronic colitis model, a preclinical, experimental model of Crohn’s disease (da Cunha et al., 2020). However, only the recombinant strain expressing Hsp65 was tested *in vivo*, and its benefits, compared with the wild-type strain, have not been assessed. Another study used a DSS-induced IBD mouse model to compare the effects of the NCDO2118 wild-type strain and a recombinant strain producing 15-lipoxygenase-1 (Carvalho, 2016). Finally, cloning of the Growth Differentiation Factor 11(GDF11) protein in this strain enhanced its anti-inflammatory against mucositis compared to the wild-type strain (Américo et al., 2023). However, this work was carried out in a different inflammation context, i.e., mucositis, an inflammation of the mucous membranes of patients undergoing chemotherapy.

Recently, Alves et al. (2023) explored the protective effects of the wild-type strain NCDO2118 in a DSS-induced colitis mouse model. Interestingly, the effects of administration of *L. lactis* NCDO2118 pExu: *empty* observed here on clinical parameters, secretory IgA, permeability, and production of IL10 and IL6, among others, are in agreement with those reported by the above authors when using the NCDO2118 wild-type strain.

One major difference with our study is the partial preservation of weight loss in the presence of the NCDO2118 wild-type strain (Alves et al., 2023). However, it is essential to note that this difference may be attributed to different experimental models of inflammation used between the two studies. Notably, while Alves et al. (2023) induced inflammation and treated mice with bacteria during the same time over 7 consecutive days, our experimental model included a pretreatment of mice with bacterial formulations for 5 consecutive days, followed by 10 days without treatment, and then by a period of 7 days of DSS exposure to induce inflammation. Our experimental protocol was designed to evaluate the preventive effects of recombinant *L. lactis* strains in the context of UC, while the protocol used by Alves et al. (2023) was conducted to test the curative effects of the wild-type NCDO2118 as a treatment in UC.

We noted, however, that administration of NCDO2118 pExu:*empty* induced the expression of *Ocln* and decreased the production of sIgA and IL10 in healthy animals. These results were similar to those reported by De Jesus et al. (2022), and Do Carmo et al. (2020). Whether this is due to the plasmid or the properties of the strain remains unknown. These results confirm, nevertheless, the protective effect of strain NCDO2118 in another moderate DSS-induced colitis mice model and indicate that the pExu: *empty* plasmid does not appear to interfere with its health properties. The administration of *L. lactis* NCDO2118 pExu:*p62* ameliorated colon length, MPO activity, and secretory IgA levels. The number of goblet cells, notably, was higher than that found in the other groups of DSS-treated mice and similar to that in healthy mice. Goblet cells are key players in protecting intestinal mucosa (Pelaseyed et al., 2014). Their preservation might be one of the mechanisms associated with controlling colitis development by probiotics (Alves et al., 2023).

Our results suggest that the protective effects of NCDO2118 pExu:*p62* are mediated via goblet cell preservation. Maintenance of this key component of the gut barrier opens promising perspectives. Indeed, patients with intestinal inflammation exhibit alterations in the structure and biosynthesis of mucin due to reduced expression of mucin genes or goblet cells number (Strous and Dekker, 2008; Bankole et al., 2021; Wang et al., 2021). Autophagy was shown to prevent intestinal inflammation and alleviate colitis in mice treated with 2,4,6-trinitrobenzene sulfonic acid (TNBS; Macias-Ceja et al., 2017). Tiwari et al. (2021) observed that autophagy was required when mucin biosynthesis was higher in goblet cells during the endoplasmic reticulum stress and apoptosis induction. According to them, autophagy is essential for goblet cells to survive during high mucin biosynthesis, thereby regulating cellular homeostasis. The importance of autophagy was also reported by Huang et al. (2018) during intestinal endoplasmic reticulum stress. p62 protein is present in all kinds of cells and is involved in cell autophagy and immune system regulation. In the autophagy pathway, kelch like ECH associated protein 1 and nuclear factor erythroid 2 related factor 2 (Keap1 Nrf2) pathway is activated due to a dysregulation as an inflammation associated with human diseases such as IBD, for example (Moscat and Diaz-Meco, 2012; Sabbieti et al., 2015). The induction of Nrf2 can bind to ubiquitinated protein, leading to the degradation of autophagosomes (Jain et al., 2010; Ichimura et al., 2013; Wong et al., 2017; Bartolini et al., 2018).

The analysis of inflammatory parameters further revealed that *L. lactis* NCDO2118 pExu:*p62* also prevented the DSS-mediated induction of the expression of *Tnf* and *Ifn*g genes, coding for pro-inflammatory cytokines. In line with this, Cecarini et al. (2020) demonstrated that the neuroprotective effect of recombinant *L. lactis* MG1363 pExu:*p62* can be associated with its ability to modulate the TNF and IFNγ pro-inflammatory cytokines. According to Nakase et al. (2022), these cytokines are significant mediators of colitis progression and are highly secreted in the gut mucosa of UC patients. The over-expression of these pro-inflammatory cytokines is associated with increased intestinal epithelial cell death, leading to increased intestinal barrier permeability and aggravating inflammation. The genome (Oliveira et al., 2014) and proteomic (Da Silva et al., 2019) analysis was already reported for wild-type *L. lactis* NCDO2118. However, the effects of this strain on the healthy or unhealthy intestinal microbiota were never mentioned. Our work demonstrated no side effects of recombinant *L. lactis* NCDO2118 pExu:*empty* or pExu:*p62* on the microbiota diversity and composition. We conclude, thus, that the absence of microbiota modification could have been caused by low-dose DSS induction colitis, which leaded to moderate inflammation. According to Park et al. (2021), microbiota alteration depends on the dose and duration of exposure. This study a protective effect of the p62 protein delivered by *L. lactis* NCDO2118 against mice with moderate DSS-induced colitis was observed and characterized. Indeed, while both strains of *L. lactis*, with or without the p62 coding insert, mitigated the histopathological score, the crypt depth, and the permeability, only *L. lactis* with the p62 coding insert, restored goblet cells, MPO activity, sIgA, as well as *Tnf* and *Ifn*g expression. Research efforts will be necessary to elucidate better the autophagy pathway and immunological mechanisms of the p62 protein in colitis prevention.

Our study has some limitations that should be improved in future works. First, new doses of DSS and new IBD induction protocols (acute versus chronic colitis) should be tested to evaluate better the impact of *L. lactis* expressing p62 on these models, using different approaches for the treatments and prevention. Moreover, one should evaluate the expansion of immune cells (regulatory T cells, dendritic cells, macrophages) via flow cytometry to evaluate the impact of p62 on the host’s local and systemic immune response. Furthermore, more studies should be carried out to evaluate the beneficial effect of p62 on the intestinal microbiota.

Despite these limitations, our results demonstrated that *L. lactis* NCDO2118 harboring a plasmid encoding p62 protected the intestinal mucosa in moderate DSS-induced colitis by increasing goblet cells and mucin production. It further downregulated the pro-inflammatory cytokines *Tnf* and *Ifn*g gene expression. This protective effect was more efficient than the same strain devoid of p62-encoding sequence. This suggests that local delivery of p62 by an immunomodulatory probiotic may constitute an alternative therapy to mitigate a moderate UC. This opens new avenues for developing biotherapies to potentiate the existing treatment of inflammatory diseases. Indeed, immunomodulatory probiotics, in conjunction with local delivery of bioactive proteins to the colitis site, may prolong therapy-induced remissions, as was evidenced for mixtures of probiotic bacteria (Sniffen et al., 2018).

## Data availability statement

The data present in the study are deposited in the National Center for Biotechnology Information (NCBI), Bioproject: PRJNA1043697 (https://www.ncbi.nlm.nih.gov/sra/PRJNA1043697) repository, accession numbers: SAMN38350495, SAMN38350496, SAMN38350497, SAMN38350498, SAMN38350499, SAMN38350500, SAMN38350501, SAMN38350502, SAMN38350503, SAMN38350504, SAMN38350505, SAMN38350506, SAMN38350507, SAMN38350508, SAMN38350509, SAMN38350510, SAMN38350511, SAMN38350512, SAMN38350513, SAMN38350514, SAMN38350515, SAMN38350516, SAMN38350517, SAMN38350518, SAMN38350519, SAMN38350520, SAMN38350521, SAMN38350522, SAMN38350523, SAMN38350524, SAMN38350525, SAMN38350526, SAMN38350527, SAMN38350528, SAMN38350529, SAMN38350530, SAMN38350531.

## Ethics statement

The animal study was approved by Local Animal Experimental Ethics Committee of the Federal University of Minas Gerais (CEUA-UFMG) (Protocol number 178/2022). The study was conducted in accordance with the local legislation and institutional requirements.

## Author contributions

JL: Conceptualization, Data curation, Formal analysis, Funding acquisition, Methodology, Writing – original draft, Writing – review & editing. AF: Conceptualization, Methodology, Writing – review & editing. FB: Conceptualization, Methodology, Writing – review & editing. LJ: Conceptualization, Data curation, Formal analysis, Methodology, Writing – review & editing. OV: Methodology, Writing – review & editing. LQ: Methodology, Writing – review & editing. MA: Methodology, Writing – review & editing. GC: Methodology, Writing – review & editing. RG: Methodology, Writing – review & editing. JD: Methodology, Writing – review & editing. TSi: Conceptualization, Writing – review & editing. KV: Formal analysis, Methodology, Writing – review & editing. SF: Methodology, Writing – review & editing. RS: Formal analysis, Methodology, Writing – review & editing. FM: Writing – review & editing. EF: Formal analysis, Methodology, Writing – review & editing. TSa: Writing – review & editing. AB: Writing – review & editing. MO: Formal analysis, Writing – review & editing. AF: Writing – review & editing. RC: Methodology, Writing – review & editing. FV: Writing – review & editing. YL: Writing – review & editing. GJ: Writing – review & editing. EG: Writing – review & editing. VA: Funding–acquisition, Supervision, Writing – review & editing.
